# Scale Enhancement Pyramid Network for Small Object Detection from UAV Images

**DOI:** 10.3390/e24111699

**Published:** 2022-11-21

**Authors:** Jian Sun, Hongwei Gao, Xuna Wang, Jiahui Yu

**Affiliations:** 1 School of Graduate, Shenyang Ligong University, Shenyang 110159, China; 2 School of Automation and Electrical Engineering, Shenyang Ligong University, Shenyang 110159, China; 3 China State Key Laboratory of Robotics, Shenyang Institute of Automation, Chinese Academy of Sciences, Shenyang 110016, China; 4 Department of Biomedical Engineering, Zhejiang University, Hangzhou 310058, China; 5 Innovation Center for Smart Medical Technologies & Devices, Binjiang Institute of Zhejiang University, Hangzhou 310053, China

**Keywords:** object detection, unmanned aerial vehicles, small objects, feature fusion

## Abstract

Object detection is challenging in large-scale images captured by unmanned aerial vehicles (UAVs), especially when detecting small objects with significant scale variation. Most solutions employ the fusion of different scale features by building multi-scale feature pyramids to ensure that the detail and semantic information are abundant. Although feature fusion benefits object detection, it still requires the long-range dependencies information necessary for small objects with significant scale variation detection. We propose a simple yet effective scale enhancement pyramid network (SEPNet) to address these problems. A SEPNet consists of a context enhancement module (CEM) and feature alignment module (FAM). Technically, the CEM combines multi-scale atrous convolution and multi-branch grouped convolution to model global relationships. Additionally, it enhances object feature representation, preventing features with lost spatial information from flowing into the feature pyramid network (FPN). The FAM adaptively learns offsets of pixels to preserve feature consistency. The FAM aims to adjust the location of sampling points in the convolutional kernel, effectively alleviating information conflict caused by the fusion of adjacent features. Results indicate that the SEPNet achieves an AP score of 18.9% on VisDrone, which is 7.1% higher than the AP score of state-of-the-art detectors RetinaNet achieves an AP score of 81.5% on PASCAL VOC.

## 1. Introduction

UAVs have the advantages of low operational cost, high mobility, and multiple viewpoints, thus promoting the application of drone object detection [1,2] in many fields, such as power line detection [3], precision agriculture [4], and environmental monitoring [5,6]. Under the positive influence of maturity of hardware devices and the availability of training datasets, deep learning has achieved unprecedented success because of its impressive ability to learn representation from data. At present, UAV image detection algorithms are generally based on convolutional neural networks (CNNs), such as ResNet [7], DenseNet [8], and ConvNet [9]. Due to CNNs’ strong local perception and inductive biases, a series of breakthroughs have been made in computer vision tasks, such as object detection [10], semantic segmentation [11,12], human–robot interaction [13], etc. Although deep learning has made significant progress in natural image detection, aerial image detection of state-of-the-art object detectors, such as YOLO [14] and RetinaNet [15], still needs to be more satisfactory in terms of both accuracy and efficiency.

**Figure 1:**
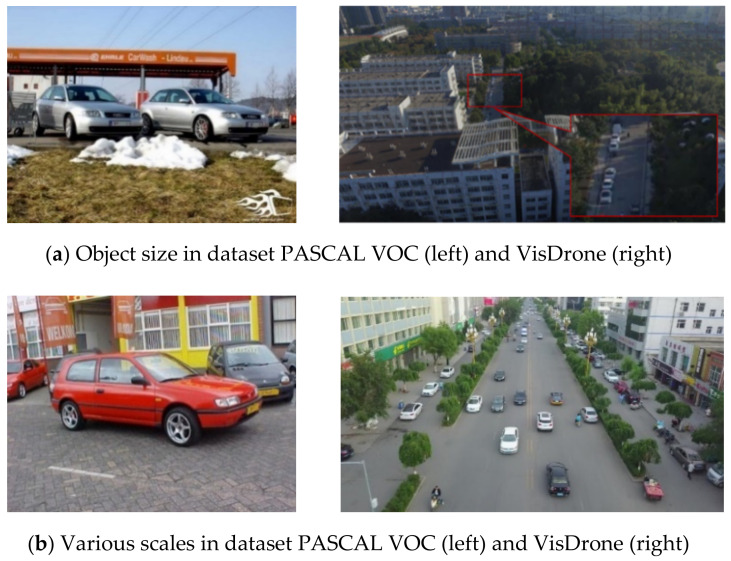
Compared with natural scene images, UAV images from VisDrone show great challenges. (**a**) Object with a small size and sparse distribution in a UAV image. (**b**) The particular perspective of the UAV makes the aerial image come in extremely varying scales.

There exist some significant differences between nature images (e.g., PASCAL VOC [16]) and UAV images (e.g., VisDrone [17]), leading to two major challenges of object detection. The first challenge is that high-resolution UAV images tend to contain small objects (area < 32^2^ pixels) and are generally sparsely distributed, as Figure 1a depicts. The features of small objects are difficult to describe because the small scale of the target is featured by fewer pixels, which is likely to cause information to gradually disperse or even vanish when they pass through a deep network. Sparse objects in images with a wide field of view are easier to be confused with complex backgrounds. Second, extreme object scale variation and special UAV perspectives can be present, as Figure 1b depicts. The UAV images of large-scale scenes are affected by the variety of altitudes and perspectives of UAVs. When UAVs shoot at lower altitudes, objects become more negligible. Objects become smaller when UAVs shoot at higher altitudes. Lengthening the perspectives also causes distant objects to become smaller. Even objects of the same class may differ several times in scale.

One way to address the challenges above is to use the cutting strategy [18,19]. The high-resolution image is dealt with as small patches and then fed separately into the network for prediction. However, such methods may require repeated computation of features, resulting in higher computation and memory requirements. In addition, multi-scale feature fusion [20,21] enriches difficulty discerning object feature representations by integrating deep and shallow features while adding less computational cost. The other line of effort aims to expand the receptive field using stacking atrous convolutions with different atrous rates or convolutional filters with different sizes [22,23], which is also an effective way to improve object detection performance. Some methods use an attention mechanism [24,25] to highlight helpful information from small targets while suppressing useless information. The attention mechanism can improve the detection performance of most existing CNN-based methods while introducing very little computation.

This paper proposes a scale enhancement pyramid network, namely SEPNet, to improve UAV image detection performance by mitigating the inconsistency in gradient computation of the adjacent layers. Our algorithm mainly consists of two core modules. We notice that the deep network is effective in detecting complex scenes. However, the deep network loses essential details in forward propagation. Although the number of network layers deepens, the receptive field becomes more significant. The single receptive field makes the detector suffer contextual limits. Based on this observation, we designed a lightweight context enhancement module (CEM) core consisting of a multi-scale dilated convolution branch and a pyramidal convolution branch. Unlike most existing methods, we combined multi-scale dilated and pyramidal convolution to model the global relationships for objects of various scales instead of artificially designed complicated decoder networks. In addition, to enhance network performance, multi-scale features are generally used to fuse information at different levels to obtain more powerful representations, and direct fusion between different levels destroys feature consistency in gradient computation, which makes features obtained after the CEM module weaken the expressive representation. We used the feature alignment module (FAM) to automatically learn the correlation between two feature layers and keep them aligned. Our SEPNet is based on one-stage detectors.

The main contributions of this paper are summarized as follows:

1. We propose a SEPNet to solve small object and multi-scale object detection difficulties in UAV images.
2. We propose the CEM to produce more salient context information by combining special groups of atrous convolutions and group convolutions and redistribution to the top of FPN, thereby improving the feature representation of objects at different scales.
3. We add the FAM that learns transformation offsets of pixels to preserve the aggregate feature space translation invariance and address the feature inconsistency issue for FPN, avoiding small objects being drowned in feature conflicts. To continue improvement, we introduce channel attention to refine pre-aggregated features while making the network focus on the target area rather than the broad background.
4. We validate the proposed two components and SEPNet on two datasets. Compared to the baseline model, RetinaNet, our component can significantly improve performance, from 21.3% to 23.5% on the VisDrone dataset. Furthermore, our SEPNet outperforms the popular detector CornerNet [26] by 1.5%.

## 2. Related Work

In this section, we briefly review the recent representative work on object detection, feature fusion architecture design, and the attention mechanism of convolutional networks.

### 2.1. Object Detection

With the development of deep learning, remarkable progress has been achieved in object detection. The mainstream object detectors based on deep learning can be divided into one-stage detectors and two-stage detectors. The significant difference between the two network architectures is that two-stage detectors first generate region proposals and then apply a convolutional network to classify and regression each region proposal. In contrast, one-stage detectors skip the proposal stage and manually set priority boxes. Two-stage methods, such as Faster RCNN [27], maintain an advantage in precision, but the speed is not satisfactory due to the need to obtain region proposals before detection. One-stage methods, such as Single Shot MultiBox Detector (SSD) [28], improve detection speed at the cost of accuracy drop. Recently, anchor-free methods were proposed. Compared to anchor-based methods, anchor-free methods replace complex anchor designs by capturing features of object centers or key points. CenterNet [29] generates heatmaps (distribution of important information in the feature map) to obtain the target center coordinates and adjust the center offset. Fully convolutional one-stage object detection (FCOS) [30], feature selective anchor-free module (FSAF) [31], and FoveaBox [32] drop prior anchor settings and directly encode and decode the bounding boxes as anchor points. This detects all positive sample points, and the positive samples point to boundary distances of the bounding box. Anchor-free methods are not constrained by predefined anchors and reduce hyperparameters and forward inference time. However, these intensive prediction tasks are prone to noise interference, resulting in many false positives.

### 2.2. Feature Fusion

Object detection in UAV images is a challenging problem due to small objects [33,34] and extreme scale variation. FPN [35] is an efficient way to alleviate the problem arising from small objects and object scale variation. In the deep network, low-level features generally lack semantic information and are rich in geometric details. In contrast, high-level features are the opposite of low-level features. FPN builds a feature pyramid to extract and fuse multi-scale features through the top-down pathway and lateral connections. The path aggregation network (PANet) [36] adds an extra bottom-up path on the top of FPN. EfficientDet [37] proposes a bidirectional feature pyramid network (BiFPN), which is a weighted bidirectional FPN used to perform fast feature fusion. Giraffedet [38] enriches multi-level contextual information through bottom-up skip-layer connection and sufficient cross-scale connection between different levels. Apart from network structure improvement, some other works [39,40] are devoted to enhancing contextual information. They generally combine multiple branches with different kernel sizes and dilated convolutions to effectively capture long-range information without reducing spatial resolution. To solve the problem of feature misalignment during high-level and low-level fusion, feature-aligned pyramid networks (FaPN) [41] achieve implicit compensation with deformable revolution to enhance feature consistency. The above methods effectively fuse different levels of semantic and location information and achieve great success but ignore the problem of feature inconsistency when dealing with different input features.

### 2.3. Attention Mechanism

The attention mechanism is recognized as a potential means to enhance deep CNNs since it allows the network to selectively focus on the most important regions of an image while ignoring the ones with irrelevant parts. Currently, attention mechanisms are prevalent in various tasks, such as machine translation [42], object detection [43], and semantic segmentation [44]. More recently, multiple attention mechanisms have provided benefits in visual studies to improve convolutional network expression ability. Squeeze-and-excitation networks (SENet) [45] are typical channel attention mechanisms. They can adaptively recalibrate channel-wise response with global contextual information by signals aggregated from feature maps. Efficient channel attention networks (ECANet) [46] employ the one-dimensional convolution layer to determine channel interaction and reduce the attention module parameters. Still, the information captured by the one-dimensional convolutional is inefficient. Selective kernel networks (SKNet) [47] apply multiple branches with different kernel sizes to adaptively adjust the receptive field, effectively increasing the flexibility of the network. Beyond channel attention, non-local neural networks (non-local) [48] deploy self-attention as a generalized global operator to capture the long-range dependencies. Non-local can effectively capture global features of spatial sequences and are more friendly for video detection. Convolutional block attention modules (CBAM) [49] and bottleneck attention modules (BAM) [50] introduce channel and spatial attention to allow the network to generate weights of different channels and spatial automatically, highlighting the location and category information of the network. Furthermore, SANet [51] propose efficient shuffle attention, which can effectively combine spatial and channel attention through shuffle units to enrich the network with deep information. In contrast, our work focuses on the correlation of channels between different levels of features to further integrate information at different scales of the feature map.

## 3. Method

**Figure 2:**
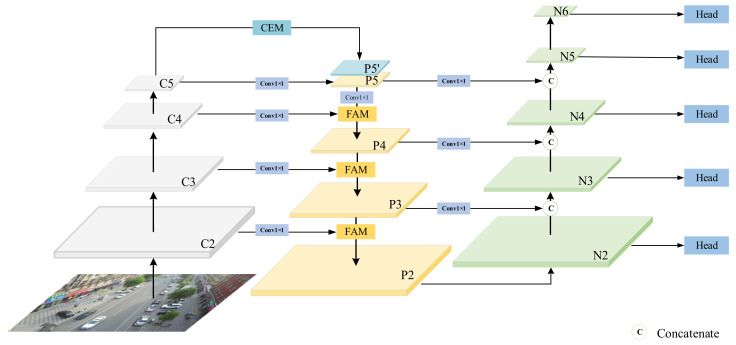
The overall architecture of the proposed SEPNet.

The overall architecture of SEPNet is shown in Figure 2. We first use ResNet to build our backbone network as the feature extractor. Each pyramidal feature map (denoted C2, C3, C4, C5) extracted by ResNet is followed by an additional 1 × 1 convolution to compress channels. Then, these feature maps are used to build a feature pyramid for multi-scale detection. We input C5 into a CEM module and concatenate it with P5 to obtain rich semantic information. We also use the FAM module to learn the correlation of pre-fused features, preventing important information from being destroyed when features are aggregated. It is worth noting that we use the concatenate operation instead of the sum operation for bottom-to-top feature fusion. After addressing top-to-bottom (denoted P2, P3, P4, P5) and bottom-to-top (denoted N2, N3, N4, N5) feature fusion, we will describe the implementation details of the main modules in the following sections.

### 3.1. Context Enhancement Module

**Figure 3:**
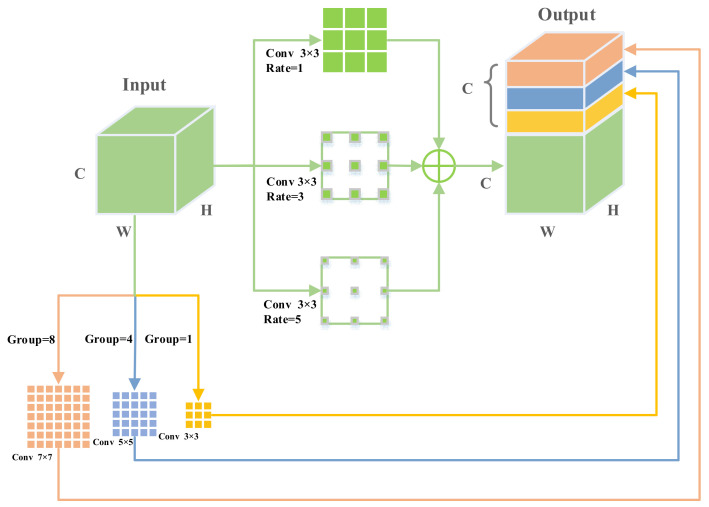
The CEM structure consists of two branches. One branch is processed by dilated convolutions with rates of 1, 3, and 5. The other is processed by grouped convolutions divided into groups 1, 4, and 8, respectively. Finally, two branches are processed by concatenating.

As we all know, with the deepening of the network layer, the features lose spatial information, and the ability to express features is weakened. In addition, due to the fixed convolution operation, the features lack the contextual information necessary for object detection at different scales. To extract high-level information, atrous spatial pyramid pooling (ASPP) [52] uses atrous convolutions of different dilation rates to capture the context at multiple scales. Although ASPP can encode multi-scale information and proves effective in semantic segmentation, we believe that the uniform resolution obtained by atrous convolution alone is not enough for UAV detection. For this reason, we are inspired by PyConv [53] and propose a context enhancement module (CEM), which aims at optimizing the deeper layer features to avoid the propagation of lost information features in FPN. CEM injects rich context information into the top of the feature pyramid network to enhance object feature representation, as shown in Figure 3.

**Figure 4:**
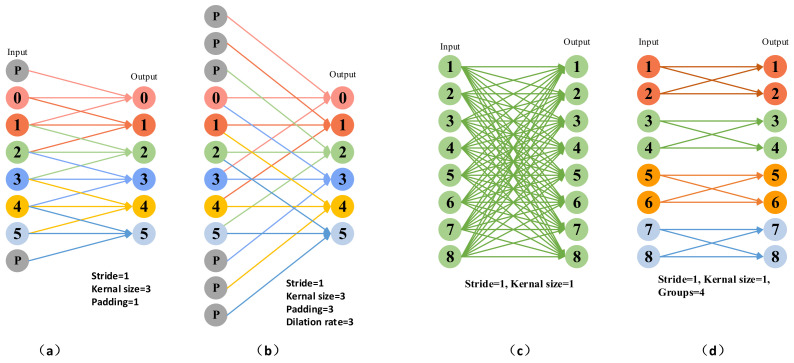
Different convolution visualization results. (**a**) is standard convolution, kernel size is 3 × 3 with padding 1, and the stride is 1. (**b**) represents atrous convolution, kernel size is 3 × 3 with dilation rates 3, padding is 3, and stride is 1. (**c**) is the standard convolution, and the kernel size is 1 × 1. (**d**) shows the grouped convolution is split into four groups.

The critical components in CEM include atrous spatial pyramid convolutions and grouped pyramidal convolutions. To better explain our CEM, we use a graph to show standard convolution, atrous convolution, and grouped convolution, as shown in Figure 4.

We use a one-dimensional expansion to demonstrate the different convolutions used in our CEM components. The 3 × 3 convolution allows for the efficient extraction of local features, and the underlying architecture is optimized for it. The 1 × 1 convolution mainly serves to integrate information between feature channels. The advantage of atrous convolution is that it can increase the receptive field without reducing the feature resolution. The characteristic of grouped convolution is that the computational complexity decreases with the number of groups increasing.

Having understood the purpose and core components of CEM, we describe it in a more rigorous mathematical formulation and explain why it is beneficial for the network. Specifically, let us first consider an input feature $X\in {\mathbb{R}}^{C\times H\times W}$, where C, H, and W indicate the channel number, spatial height, and width. CEM performs three parallel convolutions with different atrous rates to enlarge the receptive field without adding extra kernel parameters. The formula for the three parallel atrous convolutions with different atrous rates is as follows:

(1) $$O_{d}=\sum_{k=1}^{N}\sum_{a=1}^{N}D_{k,2a-1}\left(X\right),$$

In Equation (1), where $X\in {\mathbb{R}}^{C\times H\times W}$ is the input feature, $O_{d}\in {\mathbb{R}}^{C\times H\times W}$ is the output feature, where $D_{k,2a-1}\left(\cdot \right)$ means the atrous convolution, k, a denotes the filter size and the dilation rates, respectively, and N represents the number of atrous convolutions. We add three different sets of $D_{k,2a-1}\left(\cdot \right)$ to obtain the intermediate output $O_{d}$.

Considering that the atrous convolution loses detailed information, we add different groups of convolutions to supplement the different levels of detailed information. In addition, we also apply different sizes of convolution kernels to obtain different spatial resolutions, effectively alleviating object scale variation in UAV images. Grouped convolution is lightweight and efficient, adding a small amount of extra computation to improve performance. We use three levels of different kernel sizes: 3 × 3, 5 × 5, and 7 × 7, and the corresponding grouping depths are 1, 4, and 8, respectively. It can be formulated as follows:

(2) $$O_{g}=Concate\left(\left[G_{k,g}\left(X\right),G_{k,g}\left(X\right),G_{k,g}\left(X\right)\right]\right),$$

$G_{k,g}\left(\cdot \right)\in {\mathbb{R}}^{C/3\times H\times W}$ is grouped convolution, k and g correspondingly denote the filter size and the split into different groups, and $Concate\left(\cdot \right)$ means the concatenation operation. $O_{g}$ is the concatenation of grouped convolution operations of different groups.

Finally, we concatenate $O_{d}$ and $O_{g}$ to obtain semantically rich output features. The CEM formula is defined as:

(3) $$O=conv\left(Concate\left(\left[O_{g},O_{d}\right]\right)\right),$$

$conv\left(\cdot \right)$ is 1 × 1 convolution. We apply a 1 × 1 convolution to reduce the feature maps to the same as the X. Note that in this architecture, when we connect the input and output, there are multiple branching paths to obtain different levels of receptive fields. Our CEM uses a sizeable receptive field to capture semantic information and a small receptive field to capture location information. Therefore, the CEM module can effectively deal with object scale changes.

### 3.2. Feature Alignment Module

We noticed that the main reason for the poor detection of small objects in aerial image detectors is that the location information obtained by the fusion of adjacent feature layers is inaccurate, and small objects are susceptible to location deviation. To this end, we introduce the FAM to add modulated deformable convolution and channel attention based on FPN.

**Figure 5:**
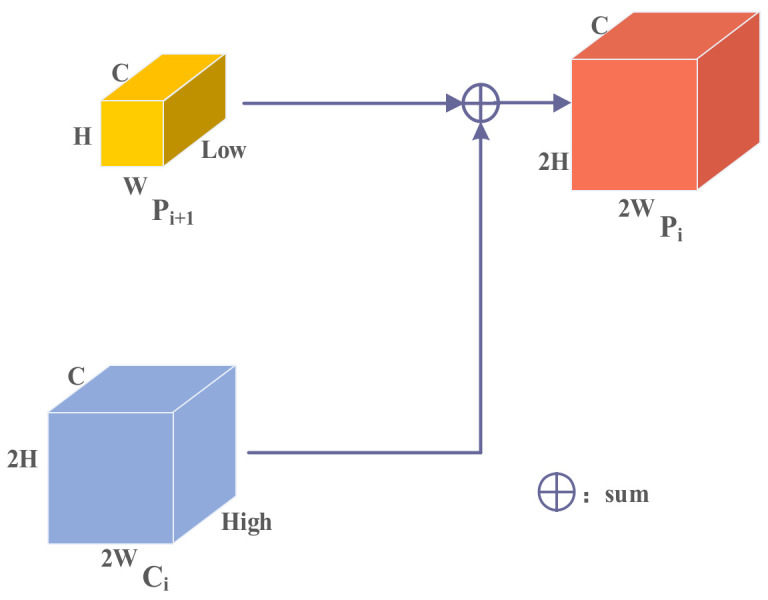
The structure of FPN.

**Figure 6:**
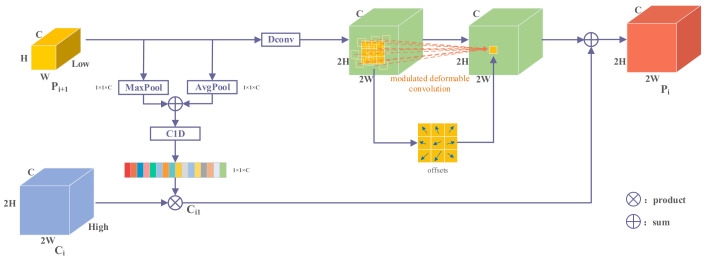
The structure of FAM.

First, let us review the FPN structure, as shown in Figure 5. In FPN, high-level features use up-sampling operations and fuse with the feature maps at low-level features, enabling the low-level feature to obtain high-level semantic information. The resulting features are naturally endowed with different levels of contextual information. However, the significant problem is that merging adjacent layer features without special processing destroys feature consistency at scale and semantic levels. We introduce the FAM module to solve this problem. The structure of FAM is shown in Figure 6.

Next, we introduce the core parts of FAM in detail. Our survey found that traditional convolution cannot make adaptive changes when adjacent features are fused due to fixed operation rules. Deformable convolutions [54] learn offsets for the convolution sampling points with freeform sampling grids, and the aim is to make the receptive field adaptively zoomed. Due to this characteristic, it is widely used for feature alignment or dealing with dense spatial transformations and can learn according to the actual scene of the data. Formally, the deformable convolution operation is defined as follows:

(4) $$Y\left(P\right)=\sum_{n=1}^{K}W_{n}\times X\left(P+P_{n}+\Delta P_{n}\right),$$

where $X\in {\mathbb{R}}^{C\times H\times W}$ is the input feature map, $Y\left(P\right)\in {\mathbb{R}}^{C\times H\times W}$ is the out feature map, and K and n refer to the size of the kernel and the index, respectively. $W_{n}$, P, and $P_{n}$ are the nth weight, indices of the center, and the nth prespecified offset, respectively. $\Delta P_{n}$ is the additional learnable offset. Since the learnable offset $\Delta P_{n}$ is typically fractional, we use the bilinear interpolation difference to obtain the position of the $\Delta P_{n}$ in the feature map.

To further enhance the feature alignment ability, modulated deformable convolution [55] adds an adjustment mechanism based on deformable convolution, which can effectively adjust the offset of the perceptual input features. The modulated deformable convolution is defined in Equation (5):

(5) $$Y\left(P\right)=\sum_{n=1}^{K}W_{n}\times X\left(P+P_{n}+\Delta P_{n}\right)\cdot \Delta m_{n},$$

where $\Delta m_{n}$ is the modulation scalar for the nth location. FAM uses modulated deformable convolution to learn offsets after the up-sampling of high-level features.

Furthermore, we pass the channel information of high-level features to low-level features through channel attention to inject the low-level features with semantic information. SENet pioneered channel attention, with consists of two parts: squeeze and excitation. SENet uses global average pooling to recalibrate the channel-wise relationship adaptively. This operation can then be expressed as:

(6) $$Y_{i}=\left(sigmoid\left(W_{2}\times ReLU\left(W_{1}F_{avg}\left(X\right)\right)\right)\right)\times X,$$

(7) $$k=\left|\frac{\log_{2}(C)}{\gamma }\right.+\left.\frac{b}{\gamma }\right|,$$

where $W_{1}$ and $W_{2}$ represent the fully connected layers, $Y_{i}\in {\mathbb{R}}^{C\times H\times W}$ is the result of the channel attention output, $F_{avg}\left(\cdot \right)$ is global average pooling, and sigmoid represents activation function and aim to normalize the data. SENet uses two fully connected layers to learn channel weights. In order to reduce the complexity of the model, dimensionality reduction operations are performed, which bring some negative effects. We use one-dimensional convolution of size k instead of full connection, and k represents the range of channel learning. The size of k can be obtained by Formula (7), where C is the channel number, and γ and b are the two adjustable variables in the non-linear mapping. We set γ and b to 2 and 1, respectively.

(8) $$C_{i1}=\left(sigmoid\left(C1D_{k}\;\left(F_{avg}\;\left(P_{i+1}\right)+F_{max}\;\left(P_{i+1}\right)\right)\right)\right)\times C_{i},$$

where $C1D_{k}\left(\cdot \right)$ is the one-dimensional convolution of size k, $C_{i}\;\in {\mathbb{R}}^{C\times 2H\times 2W}$ is a high-level feature, $P_{i+1}\;\in {\mathbb{R}}^{C\times H\times W}$ is a low-level feature, and $F_{max}$ is global max pooling. $C_{i1\;}\in {\mathbb{R}}^{C\times 2H\times 2W}$ is the result of the attention output. Different from FPN, our FAM uses learnable deconvolution to enlarge feature map resolution instead of up-sampling and then uses modulated deformable convolution adaptively learned feature offset to align spatial features. FAM method can be written as:

(9) $$P_{i}=Y\left(Deconv\left(P_{i+1}\right)\right)+conv(C_{i1}),$$

where $C_{i}$ and $P_{i+1}$ are the inputs of two adjacent feature layers, $Y\left(\cdot \right)$ represents the modulated deformable convolution, $P_{i}\in {\mathbb{R}}^{C\times H\times W}$ is the output of FEM, and $Deconv\left(\cdot \right)$ means deconvolution. We perform the $Deconv\left(\cdot \right)$ operation on the low resolution $P_{i+1}$ to obtain higher-resolution features. FAM suppresses inconsistencies in gradient computation by modulating deformable convolution before feature aggregation. In addition, we obtain the channel attention of high-level semantic features to low-level features.

## 4. Experiments

In this section, we first introduce the dataset and implementation details. Then, we conduct ablation studies to prove the effectiveness of each model. In addition, we compare the proposed SEPNet with other methods and provide detailed and abundant analyses of the experiments provided to understand our framework better. Finally, we present a visual analysis of the detection results, which shows that the problems of small objects and significant scale changes in SEPNet are indeed alleviated.

### 4.1. The Dataset and Evaluation Metrics

To evaluate the proposed method, we conduct quantitative experiments on aerial image datasets VisDrone 2019 and PASCAL VOC 2007/12, respectively.

VisDrone2019: The drone platform acquires the dataset and contains different weather and light conditions representing common scenarios in our daily lives. The image scale of the dataset is approximately 2000 × 1500 pixels. The VisDrone 2019 has 10 object classes and consists of 6471 training images, 548 validation images, and 1610 testing images.

PASCAL VOC2007/12: The PASCAL VOC 2007/12 is the standard object detection dataset with 20 object classes and includes 22,136 training images and 5000 validation images. We train models on PASCAL VOC2007/12 train-val sets and report results on the VOC2007 test set with a total of 4952 images.

For VisDrone, we follow the standard MS COCO [56] protocol where average precision (AP) is measured by averaging multiple intersection over union (IoU) [57] thresholds to evaluate the performance. We use AP, AP50, AP75, APs (area < 32^2^ pixels), APm (32^2^ < area < 96^2^ pixels), and APl (area > 96^2^ pixels) as the metrics to measure precision; AP50 and AP75 are computed at the single complete intersection over union (CIoU) [58] threshold 0.5 and 0.75 overall categories. For PASCAL VOC, we use mean of average precision (mAP) to evaluate our model, and the CIoU threshold is set to 0.5.

### 4.2. Data Augmentation

**Figure 7:**
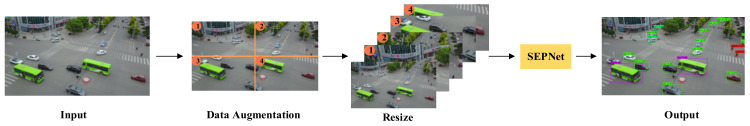
In the data augmentation method, input images are uniformly divided into 4 patches without overlapping.

Data augmentation only processes the input image without changing the network structure or adding extra parameters. Therefore, it hardly adds extra computation and can be applied to various computer vision tasks. In SEPNet, we use a combination of geometric augmentations (such as horizontal flipping, random cropping of the images, resizing, etc.) and photometric augmentations (such as brightness adjustment, contrast adjustment, saturation adjustment, and adding noise to images) in data augmentation. In addition, we follow the training practices below: Most images are large in VisDrone, resulting in the disappearance of small target features after down-sampling by the deep network. Therefore, input images are uniformly divided into four patches without overlapping during training and inference. Each patch is fed into the network for further precise detection. Meanwhile, the original images are also forwarded to the network to detect large objects and prevent the clipped target from being undetectable. Finally, the detection results of each patch and the original image are combined to obtain the final result. The image is divided into a four patches strategy, as shown in Figure 7.

### 4.3. Implementation Details

For most experiments, we trained and evaluated the models on a machine with 1 NVIDIA RTX 3090 GPU, CUDA 11.1, and implemented the proposed SEPNet on Pytorch 1.70. Our experiments were conducted on VisDrone and PASCAL VOC datasets, respectively. We selected object detectors RetinaNet as our baseline model, and ResNet pretrained in ImageNet was used as the backbone.

In the training phase, we applied the stochastic gradient descent (SGD) optimizer with a batch size of 32 images per GPU. Weight decay and momentum were set to 0.0005 and 0.9. We trained our models for 150 epochs, with the initial learning rate set to 0.001, decaying by 10 separately at epochs 90 and 120, and the resolution size of the input image was set to 800 × 800. On PASCAL VOC, the epochs were set to 200, and the learning rate was set to 0.005 and decreased 0.1 times after the 90th and 150th rounds.

The loss function for classification was the focal loss [15], and the smooth L1 [59] was used for regression. The overall training objective was:

(10) $$Loss=\frac{1}{N_{POS}}\sum_{i}L_{cls}^{i}+\frac{1}{N_{POS}}\sum_{j}L_{reg}^{j},$$

where N is the number of matched positive samples, $L_{cls}^{i}$ and $L_{reg}^{j}$ stand for the classification loss and regression loss, respectively, $N_{POS}$ is the number of positive samples, i are all positive and negative samples, and j are all positive samples. For data augmentation, we adopted the same method as that in Section 4.2. During the inference process, bounding box regression was the crucial step. IoU measures the positional relationship between the predicted box and the ground-truth box. However, IoU has the problems of slow convergence and inaccurate regression when detecting small objects. Therefore, IoU was replaced by CIoU loss. Unlike IoU, CIoU considers bounding box overlap size, center point distance, and aspect ratio. IoU is defined as shown in equation:

(11) $$IoU=\frac{\left|A\right.\cap \left.B\right|}{\left|A\right.\cup \left.B\right|},$$

where A and B are the ground-truth box and predicted box. Penalty term can be represented as:

(12) $$R_{CIoU}=\frac{\rho^{2}\left(b,b^{gt}\right)}{c^{2}}+\alpha \upsilon ,$$

where b and $b^{gt}$ are the central points of the predicted box and ground-truth box, $\rho \left(\cdot \right)$ denotes the Euclidean distance, and c is the diagonal length of the smallest enclosing box covering the two boxes. v measures the consistency of the aspect ratio as follows:

(13) $$\nu =\frac{4}{\pi^{2}}{\left(\mathrm{arc}\tan\frac{w^{gt}}{h^{gt}}-\mathrm{arc}\tan\frac{w}{h}\right)}^{2},$$

where $w^{gt}$ and $h^{gt}$ are the width and height of the ground-truth box, and w and h denote the width and height of the predicted box. α is a positive trade-off parameter, as seen in Equation (14):

(14) $$\alpha =\frac{\upsilon }{\left(1-IoU\right)+\upsilon }.$$

The loss function can be defined as:

(15) $$CIoU=1-IoU+R_{CIoU}.$$

### 4.4. Ablation Study

In this section, we conducted ablation experiments to analyze the effectiveness of each component and compared them with the baseline model RetinaNet on the VisDrone dataset.

We gradually applied data augmentation, CEM, and FAM to the baseline model to verify its effectiveness and compare it with the baseline model. At the same time, we analyzed why each component can improve network performance.

Ablation study results on the VisDrone test set are shown in Table 1, and the IoU threshold for non-maximum suppression was set to 0.5. We can observe that our method significantly improved object detection performance, especially for small objects. Specifically, data enhancement saw a 1.1% AP increase without introducing additional parameters; CEM and FAM improved the baseline method by 0.6% AP and 0.5% AP and introduced 2.3M and 2.1M parameters, respectively. Combining three strategies improved baseline model detection performance from 21.3% to 23.5% AP when using ResNet-50 as the backbone. In addition, our strategy significantly improved small object detection by 2.2% AP, only adding 4.4M parameters. The above experimental results demonstrate that the CEM component can effectively supplement contextual information of deep networks to improve scale variation detection performance. It was also verified that the FAM embedded in the baseline model is helpful for the fusion of adjacent features and effectively improves the detection results of small objects. At the same time, our data augmentation strategy can effectively avoid the problem of losing small object information during down-sampling, so it can improve the detection accuracy of small objects.

**Figure 8:**
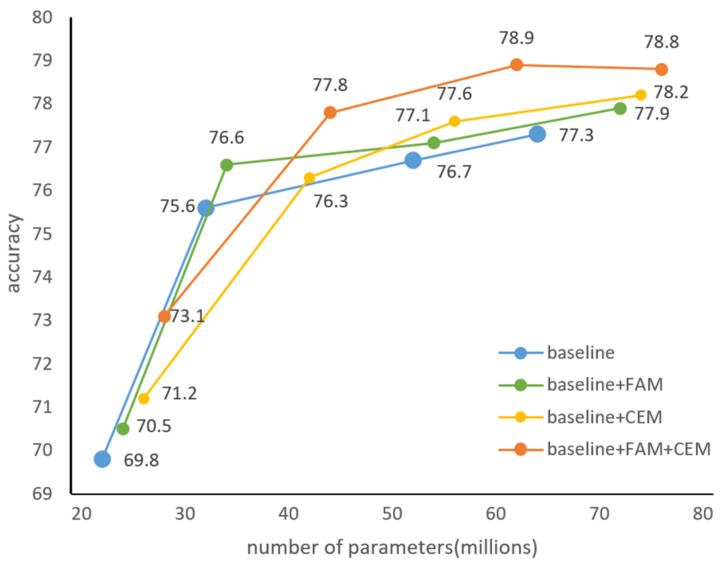
Analysis of the relationship between accuracy and number of parameters in the PASCAL VOC test set.

To verify the generalization ability of proposed method, two components were trained and tested on the PASCAL VOC dataset. We gradually added each component to the baseline model and analyzed the accuracy and number of parameter relationships using ResNet-19, ResNet-50, ResNet-101, and ResNet-152 as the backbone network, respectively. The experimental results are shown in Figure 8.

In the PASCAL VOC test set, for ResNet-19 as the backbone network, the detection accuracy was increased by 1.4% and 0.7% after adding CEM and FAM components, respectively. Combining the use of CEM and FAM components, accuracy was increased by 3.3%, and the number of parameters was increased by 4.4M. For ResNet-50 as the backbone network, combining two components improved baseline model detection performance from 75.6% to 77.8%. For ResNet-101 as the backbone network, each component also improved the model’s accuracy. It is worth noting that when the backbone network was switched from ResNet-101 to ResNet-152, combining the two components into the baseline model, the accuracy no longer increased.

These experiments prove that our two components achieve significant improvements by introducing fewer additional parameters and can adapt to different datasets, indicating their effectiveness and generality.

### 4.5. Comparisons with Other Methods

Regarding VisDrone and PASCAL VOC, we compared the performance of our SEPNet with other popular one-stage detectors and two-stage detectors. The experimental results are shown in Table 2.

In this experiment, we used the training set of VisDrone for training and the test set for validation. Table 2 shows the comparison of our proposed method with some current popular methods. Our SEPNet outperformed Cascade R-CNN and Light-RCNN by 2.8% and 2.4%, respectively. Compared with existing one-stage methods, our SEPNet outperformed CornerNet by 1.5%, 0.7%, and 0.9% on AP, AP50, and AP75, respectively.

In addition to the contrast experiments on VisDrone2019, we also conducted experiments on PASCAL VOC to verify the generalization of SEPNet. We reported results on the PASCAL VOC test set. The experimental results are shown in Table 3.

We compared our SEPNet with popular detectors in the PASCAL VOC test set. The experimental results show that our SEPNet outperforms the advanced one-stage detection algorithms DSSD and CenterNet by 2.9% and 0.8%, respectively. Compared to the two-stage algorithms Faster R-CNN and R-FCN, our SEPNet also increased by 5.1% and 1%, respectively. The experimental observations on the PASCAL VOC test dataset maintained a consistent improvement with the experimental results on the VisDrone dataset, which demonstrates that our method has similar generalization ability to other datasets and can be applied to different scenes.

**Figure 9:**
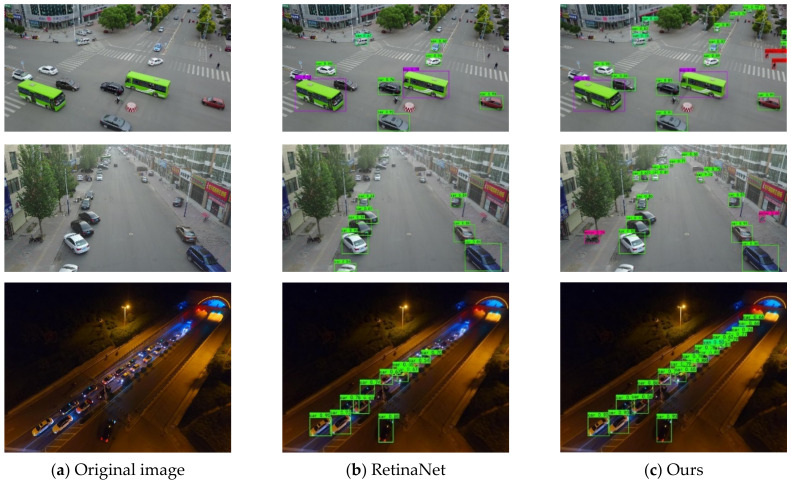
Visualization of detection results on VisDrone. Our SEPNet predicts more refined boundaries and learns more detailed information.

**Figure 10:**
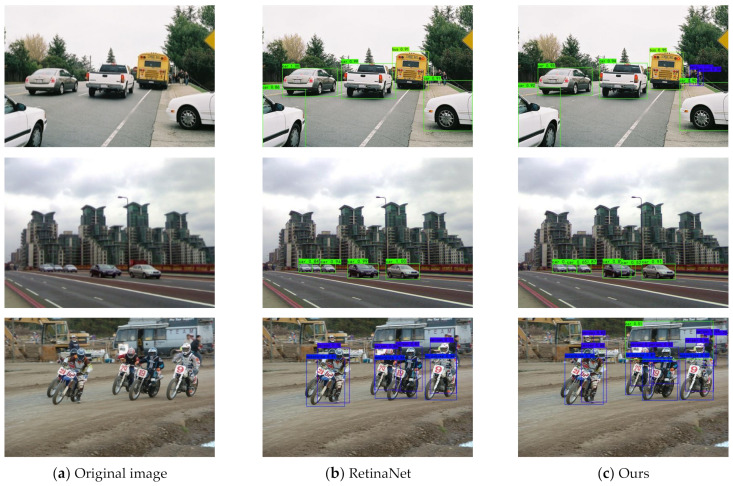
Continuation. Other visualization examples of detection results on PASCAL VOC.

To further demonstrate the effectiveness of the proposed SEPNet more intuitively, we present some visualization results in Figure 9 and Figure 10. We compared our methods with RetinaNet. RetinaNet can only detect large objects close to the camera and misses small objects far away. Compared with RetinaNet, we proposed that SEPNet could detect not only large objects in the image but also small objects far from the camera. This indicates that our SEPNet can capture objects of different scales more accurately while paying more attention to the small object region rather than the surrounding background. It can be seen from the visualization results that SEPNet can solve the problem of missed detection of small objects well. It can also be seen that SEPNet can adapt well to object scale changes and improve detection accuracy.

## 5. Conclusions

This paper proposes a one-stage scale enhancement pyramid network (SEPNet) to solve small object and extreme scale variation problems in UAV images. The proposed method consists of two main core components: CEM maintains deep features with rich contextual information, avoiding the loss of small target information and FAM addresses the lack of effective communication between adjacent features. Our results show that the proposed components offer significant improvements. Furthermore, our SEPNet exhibits good generalization in different datasets. In future work, we will focus on designing lightweight structures for models to be deployed into embedded devices.

## Figures and Tables

**Table 1:** Ablation study results on VisDrone. RetinaNet was selected as the baseline, and we gradually added our components to the baseline to verify the effectiveness of each component. “DA” represents the data augmentation.

Backbone	DA	CEM	FAM	AP	AP_s_	AP_m_	AP_l_	Params
ResNet-50	Baseline			21.3	11.2	32.2	47.5	37.8 M
	√			22.4	12.3	32.9	48.1	37.8 M
		√		21.9	11.8	32.7	48.3	40.1 M
			√	21.8	11.9	32.5	47.7	39.9 M
	√	√	√	23.5	13.5	33.8	48.9	42.2 M

**Table 2:** Comparison of our method with other state-of-the-art methods for object detection on the VisDrone test set.

Method	Backbone	AP	$AP_{50}$	$AP_{75}$
**One-stage:**				
RetinaNet [15]	Res101	11.8	21.4	11.6
CenterNet [29]	ResNext-101-64x4d	14.2	19.3	15.5
RefineDet512 [60]	VGG-16	14.9	28.8	14.1
FPN [35]	VGG-16	16.5	32.2	14.9
CornerNet [26]	Hourglass-104	17.4	34.1	15.8
**Two-stage:**				
Cascade R-CNN [61]	ResNet101	16.1	31.9	15.0
Light-RCNN [62]	ResNet101	16.5	32.8	15.1
**Ours:**				
SEPNet	ResNext-101	18.9	34.8	16.7

**Table 3:** Results on the PASCAL VOC test set. Comparison with the other state-of-the-art methods, ours is better.

Method	Backbone	Train	Test	mAP/%
**One-stage:**				
RFBNet [63]	VGG16	VOC2007 + 2012	VOC2007	76.8
SSD300 [28]	VGG16	VOC2007 + 2012	VOC2007	77.1
SSD512 [28]	VGG16	VOC2007 + 2012	VOC2007	78.5
DSSD [64]	ResNet-101	VOC2007 + 2012	VOC2007	78.6
CenterNet [29]	ResNet-101	VOC2007 + 2012	VOC2007	78.7
YOLO v3 [65]	Darknet-53	VOC2007 + 2012	VOC2007	79.4
FCOS [30]	ResNet-101	VOC2007 + 2012	VOC2007	80.1
CenterNet [29]	DLA	VOC2007 + 2012	VOC2007	80.7
**Two-stage:**				
Fast R-CNN [59]	VGG16	VOC2007 + 2012	VOC2007	70.0
Faster R-CNN [27]	ResNet-101	VOC2007 + 2012	VOC2007	76.4
R-FCN [66]	ResNet-101	VOC2007 + 2012	VOC2007	80.5
**Ours:**				
SEPNet	ResNet-101	VOC2007 + 2012	VOC2007	81.5

## Data Availability

The data presented in this study are available on request from the corresponding author.
